# Effects of Levodopa on quality of sleep and nocturnal movements in Parkinson’s Disease

**DOI:** 10.1007/s00415-021-10419-7

**Published:** 2021-02-05

**Authors:** Eva Schaeffer, Thomas Vaterrodt, Laura Zaunbrecher, Inga Liepelt-Scarfone, Kirsten Emmert, Benjamin Roeben, Morad Elshehabi, Clint Hansen, Sara Becker, Susanne Nussbaum, Jan-Hinrich Busch, Matthis Synofzik, Daniela Berg, Walter Maetzler

**Affiliations:** 1 Department of Neurology, Christian-Albrecht-University Kiel, Arnold-Heller-Straße 3, Kiel, Germany; 2 Department for Neurology, SHG-Kliniken Sonnenberg, Saarbrücken, Germany; 3 Department of Neurodegeneration, Hertie Institute for Clinical Brain Research, University of Tübingen, Tübingen, Germany; 4 German Center for Neurodegenerative Diseases (DZNE), Tübingen, Germany; 5 Studienzentrum Stuttgart, IB Hochschule für Gesundheit und Soziales, 70178 Stuttgart, Germany

**Keywords:** Parkinson Disease, Sleep, Levodopa, Hypokinesia

## Abstract

**Background:**

Sleep disturbances are common in Parkinson’s Disease (PD), with nocturnal akinesia being one of the most burdensome. Levodopa is frequently used in clinical routine to improve nocturnal akinesia, although evidence is not well proven.

**Methods:**

We assessed associations of Levodopa intake with quality of sleep and perception of nocturnal akinesia in three PD cohorts, using the Parkinson’s Disease Sleep Scale (PDSS-2) in two cohorts and a question on nocturnal immobility in one cohort. In one cohort also objective assessment of mobility during sleep was performed, using mobile health technology.

**Results:**

In an independent analysis of all three cohorts (in total *n* = 1124 PD patients), patients taking Levodopa CR reported a significantly higher burden by nocturnal akinesia than patients without Levodopa. Higher Levodopa intake and MDS-UPDRS part IV scores (indicating motor fluctuations) predicted worse PDSS-2 and higher subjective nocturnal immobility scores, while disease duration and severity were not predictive. Levodopa intake was not associated with objectively changed mobility during sleep.

**Conclusion:**

Our results showed an association of higher Levodopa intake with perception of worse quality of sleep and nocturnal immobility in PD, indicating that Levodopa alone might not be suitable to improve subjective feeling of nocturnal akinesia in PD. In contrast, Levodopa intake was not relevantly associated with objectively measured mobility during sleep. PD patients with motor fluctuations may be particularly affected by subjective perception of nocturnal mobility. This study should motivate further pathophysiological and clinical investigations on the cause of perception of immobility during sleep in PD.

## Introduction

Sleep disorders are among the most important symptoms in Parkinson’s Disease (PD), affecting up to 90% of patients and having a considerable impact on quality of life. Sleep quality in PD can be affected by a variety of different sleep disturbances, which are important to distinguish, as they require different treatment strategies. Among the most frequent and burdensome is nocturnal akinesia [1, 2]. In daily clinical routine, an increase of Levodopa at night, in particular of Levodopa Continuous Release (CR), is frequently used when patients report nocturnal akinesia. However, evidence supporting the use of Levodopa for improving sleep quality and nocturnal akinesia is still weak. Results were derived mainly from small studies conducted many years ago [3–5] with only one cross-over, double-blind trial for Levodopa CR [6]. As a consequence, the MDS Task Force on Non-Motor symptoms has rated the evidence of Levodopa CR for improving sleep in PD as insufficient [7, 8].

Another short-coming of the currently available clinical trials on Levodopa CR is the sole use of self-reported questionnaires assessing only subjective perception of (impaired) sleep in PD patients. Fortunately, in recent years, it has become possible to measure sleep aspects objectively with new technology [2, 9, 10].

To evaluate the effects of Levodopa and specifically the frequently used Levodopa CR on sleep, we first compared subjective rating of sleep quality in a large dataset of *n* > 1100 patients from three independent PD patient cohorts, allowing to stratify both an exploration cohort and two validation cohorts. Then, we explored the use of mobile health technology to compare effectively occurring movements during sleep with subjective ratings of nocturnal akinesia as well as with overall sleep quality rating, related to Levodopa intake, in one of our cohorts.

## Methods

### Cohorts

This study presents data from three independent cohorts. A total of *n* = 1124 patients were recruited, with *n* = 56 from cohort A, *n* = 44 from cohort B and *n* = 1024 from cohort C. All participants filled out semi-quantitative questionnaires to assess aspects of sleep. Moreover, 34 individuals from cohort A participated in an objective measurement of nocturnal movements using mobile devices.

#### Cohort A

The Training-PD study [11] was set up to evaluate the effects of different forms of training in PD patients. For this analysis, only data of the baseline assessment (cross-sectional) were used for data analysis. Fifty-six PD patients from the outpatient clinic of the department of Neurodegeneration at the University of Tübingen agreed to participate in the sleep sub-study of the Training-PD study and filled out the Parkinson’s Disease Sleep Scale (PDSS-2). Of those, 34 patients additionally agreed to wear a mobile device for the detection of nocturnal movements. Moreover, seven healthy controls took part in the assessment of nocturnal movements. The study was approved by the ethics committee of the Medical Faculty of the University of Tübingen (112/2015BO2) and written informed consent was obtained from all participants. Inclusion criteria were: (1) diagnosis of PD according to the UK brain bank criteria and (2) Hoehn and Yahr score ≤ 2.5. Healthy controls were recruited using public notices. Exclusion criteria for all study participants are comprised of: (1) the presence of relevant depressive symptoms (Beck Depression Inventory > 18 points); (2) physical status or diseases other than PD affecting physical training; (3) cognitive impairment that may interfere with study outcome (Montreal cognitive assessment, MoCA < 21); and (4) signs indicating a high risk of falls.

#### Cohort B

The aim of the ongoing, longitudinal ABC-PD study [12] is to assess disease progression of PD patients with and without (probable) amyloid beta pathology. One hundred PD patients selected according to their CSF Aß42 profile were recruited from the outpatient clinic of the department of Neurodegeneration at the University of Tübingen. Of those, data of 44 PD patients were included into the present data analysis. This study was approved by the same ethics committee (686/2013BO1) and written informed consent was obtained from all participants. Inclusion criteria for PD patients were: (1) diagnosis according to the UK brain bank criteria and (2) age between 50 and 85 years. Exclusion criteria were: (1) Beck Depression Inventory II ≥ 20 points); (2) history of clinically relevant strokes; (3) confirmed clinical diagnosis of possible or probable dementia according to the MDS Task Force criteria [13]; and (4) deep-brain stimulation surgery performed or planned.

In both studies, standardized interviews were performed, including the collection of information regarding disease duration and medication. The motor part of the Movement Disorder Society Unified Parkinson’s Disease Rating Scale (MDS-UPDRS III) was used to assess motor function in the “On”-state. Part IV was used to rate patients’ perception on motor complications.

#### Cohort C

To improve in-house patient care, the Neurological Department of the Saarland Heilstätten GmbH Clinic Sonnenberg, Saarbrücken performed an anonymous survey in 1084 PD patients conducting the self-developed Saarbrücken PD survey. Besides sleep disturbances, the survey asked for occurrence and severity of motor and non-motor symptoms. In addition, the current medication plan was obtained from the patients. As data collection was anonymous, no ethical approval was needed (confirmed by the Ethic Commission of the State Chamber of Medicine, Saarland).

All studies were performed in accordance with the Declaration of Helsinki.

### Subjective assessment of overall sleep quality and perception of nocturnal akinesia

All PD patients of the Training-PD and ABC-PD studies were asked to fill out the Parkinson’s Disease Sleep Scale (PDSS-2). The PDSS-2 comprises 15 questions on different aspects of sleep quality and impairment and is frequently used and recommended in research and clinical practice [14, 15]. A higher sum score reflects lower sleep quality. Question 9 of the PDSS-2, *Did you feel uncomfortable at night because you were unable to turn around in bed or move due to immobility?* was used to assess perception of nocturnal akinesia. Permission to use the PDSS-2 was obtained for both studies (https://eprovide.mapi-trust.org/).

The question *Do you have problems when turning during sleep?* (scoring range from 0, not true at all, to 10, absolutely true), from the Saarbrücken PD survey was used to assess perception of nocturnal akinesia in the Saarbrücken PD cohort.

### Objective assessment of nocturnal movements

Participation in the assessment of nocturnal movements using a CE-certified mobile medical device (DynaPort Minimod®, McRoberts, The Netherlands; 64 × 62 × 13 mm; 100 Hz sample frequency) was optional for all study participants of the Training-PD study. The device includes a 3D accelerometer. It was worn at the lower back for 6 consecutive nights at home. Raw data of all nights were pre-analyzed by the company (https://www.mcroberts.nl/) using validated algorithms [16].

The following parameters of the nighttime assessment (means of all nights) were considered most meaningful for the evaluation of nocturnal akinesia, and were thus included in the analysis. Shifts were defined as small (> 30° < *x* ≤ 40°), medium (> 40° < *x* ≤ 80°) and large (> 80° < *x* ≤ 120°).

Duration of total sleep time: total duration of night’s rest, measured in hours.

Total movement: percentage of time of night’s rest for which movement was detected.

Movement intensity (MI) (in gram)

- MI small shifts: weighted mean MI of movement duration for small shifts
- MI medium shifts: weighted mean MI of movement duration for medium shifts
- MI large shifts: weighted mean MI of movement duration for large shifts
- MI supine periods: weighted mean MI of movement duration for supine periods

Angular velocity (degree/seconds)

- Velocity small shifts: mean angular velocity of small shifts
- Velocity medium shifts: mean angular velocity of medium shifts
- Velocity large shifts: mean angular velocity of large shifts

To proof the validity of this method to assess sleep, we first compared all PD patients with a control group to look at PD-specific movement changes during sleep, followed by a comparison of the three different medication groups of PD patients.

### Statistics

The effect of Levodopa on quality of sleep and perception of nocturnal akinesia was first evaluated in the Training-PD study dataset. Second, the ABC-PD study and the Saarbrücken PD survey datasets were used for validation purposes. Third, objective assessment of nocturnal movements was analyzed in the Training-PD study. Statistics were performed with SPSS 24.0 (SPSS Inc., IBM, USA). Group comparisons were performed using the Mann–Whitney *U* for two-group comparisons and Kruskal–Wallis *H*-test for three group comparisons of non-normally distributed variables. Normally distributed variables were compared using Student’s *t* test or ANOVA. The Fisher’s exact test was used for comparison of the dichotomous parameters (gender). For comparisons of two groups, a *p* value of < 0.05, and for comparisons of three groups a *p* value of  < 0.017 was accepted as statistically significant. Multiple linear regression using was used to identify variables predicting quality of sleep and nocturnal akinesia. Correlations were performed with the Spearman’s correlation coefficient.

## Results

### Subjective assessment of quality of sleep

#### Group characteristics

Data of 56 patients from the Training-PD study and 44 patients of the ABC-PD were analyzed. Moreover, 1084 patients of the Saarbrücken PD survey completed the PD survey. Median age was 59 (41–80) in the Training-PD study, 65 (51–79) in the ABC-PD study and 72 (39–95) in the Saarbrücken PD cohort. The Training-PD study is comprised of 61% male patients, the ABC-PD study 73% and the Saarbrücken PD cohort 57%. The median MDS-UPDRS Part III sum score was 25 (9–64) in the Training-PD and 21 (5–56) in the ABC-PD study. Median disease duration was 3 years (1–20) in the Training-PD study and 4 years (1–14) in the ABC-PD study.

To evaluate the effects of Levodopa on quality of sleep, all cohorts were divided into three subgroups:

I. No Levodopa: no intake of Levodopa at all (Training-PD: *n* = 28, ABC-PD: *n* = 12, Saarbrücken: *n* = 170).
II. Levodopa: intake of Levodopa during the day (Training-PD: *n* = 20, ABC-PD: *n* = 19, Saarbrücken: *n* = 533).
III. Levodopa CR: intake of Levodopa CR in the evening (with or without intake of additional Levodopa during the day) (Training-PD: *n* = 8, ABC-PD: *n* = 13, Saarbrücken: *n* = 381).

Between the Training-PD and ABC-PD studies, respective subgroups did not significantly differ with regard to age, gender, MDS-UPDRS III score, Levodopa equivalent dosage (LEDD), or dopamine agonists intake. Group comparisons revealed a significant difference in the MDS-UPDRS IV score in both studies, with patients in the Levodopa CR subgroup scoring highest, followed by the Levodopa subgroup. In the Training-PD study, post hoc comparisons showed a significantly lower MDS-UPDRS-IV score in the No Levodopa subgroup, when compared to the other subgroups. In the ABC-PD study, the Levodopa CR subgroup had significantly higher MDS-UPDRS IV scores than the No Levodopa and Levodopa subgroups. In the Saarbrücken cohort, the No Levodopa subgroup was significantly younger than the Levodopa and Levodopa CR subgroups (Table 1).

**Table 1:** Group characteristics and subjective assessment of quality of sleep

Cohort 1: Training-PD study	(I) No Levodopa *n* = 28	(II) Levodopa *n* = 20	(III) Levodopa CR *n* = 8	*p* value
Age, ys^a^	54 (42–74)	65 (41–80)	67 (50–76)	0.06
Male gender, *n* (%)	17 (60.7%)	14 (70.0%)	4 (50.0%)	0.59
MDS-UPDRS III, pts^b^	25 (10)	29 (12)	21 (9)	0.19
MDS-UPDRS IV, pts^a^	0.0 (0–3)	0.0 (0–10)*	1.0 (0–9)**	0.002
Dopamine agonists LEDD, mg^a^	159 (0–360)	154 (0–1000)	80 (0–315)	0.61
PDSS-2 Sum Score, pts^b^	9 (6)	14 (7)*	19 (4)***^†^	< 0.001
PDSS-2 Question 9, pts^a^	0 (0–4)	0 (0–4)	1 (0–2)***	0.003

Cohort 2: ABC-PD study	(I) No Levodopa *n* = 12	(II) Levodopa *n* = 19	(III) Levodopa CR *n* = 13	*p* value
Age, ys^b^	64 (6)	67 (7)	63 (7)	0.31
Male gender (%)	10 (83.3%)	12 (63.2%)	10 (76.9%)	0.43
MDS-UPDRS III, pts^b^	21 (10)	21 (8)	28 (12)	0.14
MDS-UPDRS IV^a^	0.0 (0–3)	0.0 (0–7)	1.0 (0–17)**^†^	0.003
Dopamine agonists LEDD, mg^a^	118 (0–480)	210 (0–480)	236 (0–630)	0.49
PDSS-2 Sum Score, pts^b^	10 (6)	11 (6)	20 (9)*^††^	0.002
PDSS-2 Question 9, pts^a^	0 (0–1)	0 (0–2)	1 (0–3)**^†^	0.002

Cohort 3: Saarbrücken PD study	(I) No Levodopa *n* = 170	(II) Levodopa *n* = 533	(III) Levodopa CR *n* = 381	*p* value
Age, ys	70 (39–86)	74 (44–95)***	74 (42–90)***	< 0.001
Male gender (%)	84 (51.5%)	305 (58.1%)	226 (60.9%)	0.13
Nocturnal immobility, pts	1.5 (0–6)	3 (0–6)**	4 (0–6)***^†††^	< 0.001

*LEDD* Levodopa equivalent dosage; *MDS-UPDRS* Movement Disorder Society Unified Parkinson’s Disease Rating Scale; *p* level of significance; *PDSS-2* Modified Parkinson’s Disease Sleep Scale; *pts* points; *ys* years

*p < 0.05, **p < 0.005, ***p < 0.001 compared to (I) No Levodopa

^†^*p* < 0.5, ^††^*p* < 0.005, ^†††^*p* < 0.001 compared to (II) Levodopa

^a^Values presented as median (range)

^b^Values presented as mean (standard deviation)

(I) No Levodopa: no intake of Levodopa during day or night; (II) Levodopa: intake of Levodopa; (III) intake of Levodopa plus Levodopa Continuous Release

#### PDSS-2 group comparisons

Subgroup comparisons of the Training-PD study showed a group difference for the PDSS-2 sum score (*p* < 0.001). The No Levodopa subgroup had the lowest, and the Levodopa CR subgroup the highest sum score. The nocturnal akinesia question (question 9) revealed lower values in the No Levodopa subgroup, than in the Levodopa CR group (*p* = 0.002). Data of the ABC-PD study showed similar results, with a significant group difference for the PDSS-2 sum score (*p* = 0.002) and question 9 (*p* = 0.001). Patients of the No Levodopa subgroup demonstrated the lowest, patients of the Levodopa CR subgroup the highest sum scores (Table 1).

#### Survey questionnaire group comparisons

In the Saarbrücken cohort, the question *Do you have problems when turning during sleep?* showed significant differences among the three subgroups. The No Levodopa subgroup had the lowest (best), the Levodopa CR subgroup the highest (worst) scores (Table 1).

#### Predictors of quality of sleep and nocturnal akinesia

Using data from all individuals of the Training-PD and ABC-PD study, disease duration, disease severity (MDS-UPDRS III) and motor complications (MDS-UPDRS IV) as well as Levodopa daily dosage and Levodopa CR daily dosage were included in a multiple regression analysis, with (i) PDSS-2 sum score and (ii) PDSS question 9 as dependent variables (Table 2). MDS-UPDRS IV, levodopa daily dosage and levodopa CR daily dosage significantly predicted worse PDSS-2 sum scores and a higher score on the question 9 of the PDSS-2. MDS-UPDRS III scores and disease duration were not significant in this model (Table 2).

**Table 2:** Predictors of subjective quality of sleep and nocturnal akinesia

	*β*	SE	*t* value	*p* value
**Predictors of quality of sleep (PDSS-2 sum score)**				
Disease duration	− 0.29	0.19	− 1.55	0.13
MDS-UPDRS III	0.01	0.06	0.13	0.90
MDS-UPDRS IV	0.95	0.25	3.74	< 0.001
Levodopa daily dosage (mg)	0.01	0.00	2.65	0.009
Levodopa CR daily dosage (mg)	0.02	0.00	2.95	0.004
*R*^2^	0.35			
Corrected *R*^2^	0.31			

	*β*	SE	*t* value	*p* value
**Predictors of nocturnal akinesia (PDSS-2 question 9)**				
Disease duration	− 0.29	0.19	− 1.55	0.13
MDS-UPDRS III	0.01	0.06	0.13	0.90
MDS-UPDRS IV	0.95	0.25	3.74	< 0.001
Levodopa daily dosage (mg)	0.01	0.00	2.65	0.009
Levodopa CR daily dosage (mg)	0.02	0.00	2.95	0.004
*R*^2^	0.34			
Corrected *R*^2^	0.30			

*β* non-standardized β-regression coefficient, *SE* standard error of β-regression coefficient; *MDS-UPDRS* Movement Disorder Society Unified Parkinson’s Disease Rating Scale *p* level of significance

### Quantitative motor assessment of nocturnal movements

Thirty-four PD patients and 7 healthy controls from the Training-PD study participated in the ambulatory quantitative assessment of mobility during sleep. From the PD patients, 17 were in the No Levodopa subgroup, 13 in the Levodopa subgroup and 4 in the Levodopa CR subgroup. The following six parameters distinguished significantly between PD patients and healthy controls: duration of total sleep time, total movement, MI of small, medium and large shifts, and angular velocity of small shifts (Table 3). None of the parameters differed significantly between the No Levodopa, Levodopa, and Levodopa CR subgroups, respectively (Table 4). Moreover, no significant correlations were seen between the PDSS-2 sum score and parameters of quantitative mobility assessment during sleep. Moreover, none of the objective parameters correlated significantly with Levodopa Daily Dosage (data not shown).

**Table 3:** Quantitative motor assessment of nocturnal movements in PD vs. healthy controls

Training-PD study	PD *n* = 34	Controls *n* = 7	*p* value
**Duration of total sleep time (h)**	7.8 (6.6–12.7)	9.5 (8.7–12.8)	0.001
**Total movement (% of night’s rest)**			
Total detected movement^a^	1.8 (0.7–5.7)	2.6 (1.3–16.1)	0.020
**Movement intensity (mg)**			
Mean MI of movement duration small shifts^b^	45.4 (9.0)	61.8 (9.3)	< 0.001
Mean MI of movement duration medium shifts^b^	63.2 (11.5)	81.8 (16.6)	0.001
Mean MI of movement duration large shifts^b^	81.3 (17.0)	107.7 (13.7)	0.004
**Angular velocity (deg/sec)**			
Mean angular velocity small shifts^b^	5.7 (0.9)	4.8 (0.6)	0.011
Mean angular velocity medium shifts^b^	10.2 (9.0)	9.0 (1.7)	0.14
Mean angular velocity large shifts^a^	10.7 (8.1–19.0)	10.0 (8.2–11.4)	0.45

*deg/sec* degree per second; *h* hours; *n* number; *MI* movement intensity; *p* level of significance; *PD* Parkinson’s disease; *s* seconds; *g* gram

^a^Values presented as median (range)

^b^Values presented as mean (standard deviation)

**Table 4:** Quantitative motor assessment of nocturnal movements in PD patients with and without Levodopa

Training-PD study	(I) No Levodopa *n* = 17	(II) Levodopa *n* = 13	(III) Levodopa CR *n* = 4	*p* value
**Duration of total sleep time (h)**	7.7 (6.6–12.7)	8.1 (7.0–10.1)	7.7 (7.1–8.0)	0.50
**Total movement (% of night’s rest)**				
Total detected movement^a^	1.8 (0.7–5.7)	1.9 (1.0–2.9)	1.7 (0.9–2.7)	0.79
**Movement intensity (mg)**				
Mean MI of movement duration small shifts^b^	44.4 (9.8)	47.6 (8.4)	42.1 (7.7)	0.48
Mean MI of movement duration medium shifts^b^	60.9 (10.2)	65.8 (12.6)	62.4 (14.2)	0.58
Mean MI of movement duration large shifts^b^	80.0 (15.6)	82.8 (20.4)	82.2 (19.2)	0.95
**Angular velocity (deg/sec)**				
Mean angular velocity small shifts^b^	5.9 (9.5)	5.5 (0.7)	6.3 (0.9)	0.21
Mean angular velocity medium shifts^b^	10.0 (1.6)	10.2 (2.3)	10.4 (2.4)	0.94
Mean angular velocity large shifts^a^	10.8 (2.6)	12.2 (3.4)	10.4 (0.5)	0.56

*deg/sec* degree per second; *h* hours; *n* number; *MI* movement intensity; *p* level of significance; *PD* Parkinson’s disease; *s* seconds; *g* gram

^a^Values presented as median (range)

^b^Values presented as mean (standard deviation), (I) No Levodopa: no intake of Levodopa during day or night; (II) Levodopa: intake of Levodopa; (III) intake of Levodopa plus Levodopa Continuous Release

## Discussion

We present here data from three independent PD cohorts, all reporting a significantly worse subjective quality of sleep and a higher perception of nocturnal akinesia when receiving Levodopa CR. Higher Levodopa and Levodopa CR dosages as well as higher MDS-UPDRS part IV scores predicted higher scores of the PDSS-2 sum score and PDSS-2 question 9, indicating worse quality of sleep. However, the objective quantitative mobility assessment during sleep, conducted in a subsample, showed no significant differences between these subgroups in amount, extent or dynamics of nocturnal movements.

Treatment recommendations for nocturnal akinesia at night comprise a change in therapy to more continuous drug delivery [17, 18], including the use of long-acting dopamine agonists and pump therapies for which profound evidence exists [19–23]. In contrast, use of Levodopa CR is missing sufficient evidence although it is frequently used in clinical routine, and the current results question whether this clinical standard is an adequate therapy for nocturnal akinesia. The intercorrelations between Levodopa (CR) dosage and MDS-UPDRS IV scores as determinants of the PDSS-2 sum score and question 9 might indicate that individuals with motor fluctuations suffer from higher burden by subjective impairment of quality of sleep and perception of nocturnal akinesia, while associated higher levodopa dosages cannot sufficiently improve this situation.

Furthermore, our study raises the question why the findings of subjective assessment of sleep in our cohorts could not be reproduced in an objective quantitative mobility assessment. Interestingly, our results are comparable with a study published by Antzack et al. [24], who similarly compared subjective assessment of sleep using the PDSS-2, with objective results obtained by polysomnography. In concordance with their study, we found a significant correlation between higher Levodopa doses and subjective impairment of quality of sleep and perception of nocturnal akinesia, but no correlation with parameters of quantitative motor assessment. When comparing all PD patients to healthy controls to confirm the validity of our quantitative motor assessment, several parameters indicating reduced nocturnal mobility in PD patients were identified. PD patients showed less overall nocturnal movements and smaller movement intensity values. These observations validate our measurements, confirm previous results and strongly suggest that the method used should be able to detect subtle deviations in mobility performance during sleep [2, 10, 25]. We, therefore, conclude that our finding, i.e., the discrepancy between subjective and objective measurements, can only be explained by an altered *perception* of nocturnal movements and quality of sleep in these patients, and not by the presence of nocturnal akinesia per se. The observation that patients’ perception can differ from objective evaluation has been previously described for dyskinesias. Interestingly, while a decreased awareness especially for peak-dose dyskinesias reflecting the on-phase of the disease has been reported [26], the off-phase of the disease seems to be associated with an increased awareness [27]. Concordant with [24], in our study higher MDS-UPDRS part IV scores predicted higher scores in PDSS-2 and question 9 of the PDSS-S, indicating presence of motor fluctuations and dyskinesias in those with lower sleep quality and increased perception of nocturnal akinesia. This emphasizes, in our view, the hypothesis that the observed discrepancy between subjective quality of sleep and objectively measured mobility during sleep may be an expression of *misperception*, potentially occurring particularly in individuals experiencing, for example, off-phases. Suspected nocturnal motor off-phases should have been detected with the quantitative motor assessment tools used, however, they were not. One possible explanation could be that the altered self-perception of sleep quality and nocturnal akinesia might be an expression of non-motor fluctuations (which we are not able to measure with the technique used in this study), rather than motor off-phases per se.

The strengths of this study include the confirmation of main study results in three independent cohorts. Still, the study faces some limitations. First, all results are obtained from cross-sectional studies, therefore, our observations do not allow conclusions regarding causality. Other factors influencing quality of life, e.g., occurrence of depression or concomitant medication, have not been considered. The subjective sleep assessment included only one questionnaire. A more comprehensive sleep assessment battery incorporating other standard sleep questionnaires and even a quantitative methodology such as polysomnography to identify (additional) reasons for insomnia that could explain our findings (such as sleep apnea and periodic limb movements) would have been desirable. Moreover, data on quantitative mobility assessment during sleep were available only from one study.

In summary, our results indicate that the regular use of Levodopa or Levodopa CR to improve sleep quality or nocturnal akinesia in PD should be critically evaluated. Our data obtained from cross-sectional studies do not allow conclusions regarding causality, leaving the question open if Levodopa (CR) might have a negative effect on sleep, or patients just do not profit sufficiently enough from it. In any case our study shows that subjective burden by impaired quality of sleep and nocturnal akinesia is still under-treated in individuals receiving Levodopa. We conclude, that in clinical routine, the effect of Levodopa CR on sleep quality in PD patients should be more regularly monitored, for instance using easy-to-apply questionnaires such as the PDSS-2 before and after initiation of treatment. Other forms of continuous release drug delivery with better evidence levels should always be considered. In addition, efforts should be made to understand the discrepancy between subjective perception of nocturnal akinesia, and (the lack of) nocturnal akinesia when assessed with objective measurement tools. For this latter purpose, longitudinal sleep assessments including objective assessment techniques for the evaluation of nocturnal akinesia should be performed, with consideration of treatment effects.

## Data Availability

The datasets of this study cannot be made publicly available because of the nature of the study (as stated in the ethical proposal written for this study). Requests to access the datasets should be directed to the corresponding author.

## Data statement

The datasets of this study cannot be made publicly available because of the nature of the study (as stated in the ethical proposal written for this study). Requests to access the datasets should be directed to the corresponding author.
